# The Age for Revaccination

**Published:** 1888-06

**Authors:** 


**The Age for Revaccination.**—The Local Government Board has issued an order reducing the age limit for revaccination from fifteen to twelve years, under ordinary circumstances, and to ten years in case there be any immediate danger of small-pox.—*Lancet, February, 1888.*

